# Long-Term Follow-Up In Paroxysmal Atrial Fibrillation Patients With Documented Isolated Trigger

**DOI:** 10.3389/fcvm.2023.1115328

**Published:** 2023-07-13

**Authors:** Zefferino Palamà, Antonio Gianluca Robles, Matteo Paoletti, Martina Nesti, Ermenegildo De Ruvo, Antonio Scarà, Alessio Borrelli, Gabriele De Masi De Luca, Mariano Rillo, Leonardo Calò, Elena Cavarretta, Silvio Romano, Luigi Sciarra

**Affiliations:** ^1^ Department of Life, Health and Environmental Sciences, University of L'Aquila, L'Aquila, Italy; ^2^ Electrophysiology Unit, Casa di Cura “Villa Verde”, Taranto, Italy; ^3^ Cardiology Department, Cardiology Unit Ospedale “L. Bonomo”, Andria, Italy; ^4^ Cardiology Unit, CNR Fondazione Toscana “Gabriele Monasterio”, Pisa, Italy; ^5^ Cardiology Unit, Policlinico Casilino, Rome, Italy; ^6^ GVM Care and Research, Ospedale San Carlo di Nancy, Rome, Italy; ^7^ Department of Cardiology, Ospedale Panico, Tricase, Italy; ^8^ Department of Medical-Surgical Sciences and Biotechnologies, Sapienza University of Rome, Latina, Italy; ^9^ Cardiovascular Department, Mediterranea Cardiocentro, Naples, Italy

**Keywords:** atrial fibrillation ablation, tailored approach, supraventricular tachycardia, PVI catheter ablation, PVI only

## Abstract

**Aims:**

Supraventricular tachycardias may trigger atrial fibrillation (AF). The aim of the study was to evaluate the prevalence of supraventricular tachycardia (SVT) inducibility in patients referred for AF ablation and to evaluate the effects of SVT ablation on AF recurrences.

**Methods and results:**

249 patients (mean age: 54 ± 14 years) referred for paroxysmal AF ablation were studied. In all patients, only AF relapses had been documented in the clinical history. 47 patients (19%; mean age: 42 ± 11 years) had inducible SVT during the electrophysiological study and underwent an ablation targeted only at SVT suppression. Ablation was successful in all 47 patients. The ablative procedures were: 11 slow-pathway ablations for atrioventricular nodal re-entrant tachycardia; 6 concealed accessory pathway ablations for atrioventricular re-entrant tachycardia; 17 focal ectopic atrial tachycardia ablations; 13 with only one arrhythmogenic pulmonary vein. No recurrences of SVT were observed during the follow-up (32 ± 18 months). 4 patients (8.5%) showed recurrence of at least one episode of AF. Patients with inducible SVT had less structural heart disease and were younger than those without inducible SVT.

**Conclusion:**

A significant proportion of candidates for AF ablation are inducible for an SVT. SVT ablation showed a preventive effect on AF recurrences. Those patients should be selected for simpler ablation procedures tailored only to the triggering arrhythmia suppression.

## Introduction

Atrial fibrillation (AF) is a disease with several possible pathogenetic mechanisms, which are partially explained by the knowledge available today (1). In most cases, the trigger factor is given by the foci in the pulmonary veins (2) but in other cases, other anatomical structures or arrhythmic conditions may act as trigger (3–5). These pathophysiological notions are in contrast with the classification of atrial fibrillation, which continues to be merely temporal (first diagnosis, paroxysmal, persistent, long-standing persistent). Certainly, we can say that the weight of triggers in paroxysmal AF is more important than in persistent/permanent; on the other side, the role of the substrate is more critical in permanent/persistent AF than in paroxysmal (1). However, this cannot be established *a priori* without proper clinical and instrumental investigation, both non-invasive (through echocardiography, stress tests, 24-hour Holter-ECG recording and loop recorder) and invasive (electrophysiological study).

From these considerations, it seems logical and well-founded that there is no unique type of ablative treatment valid for any patient with AF, conversely, a tailor-made treatment could simplify the ablative treatment of patients with AF while ensuring a reduction in risk complications and a lower relapse rate (6). Indeed, it has been shown that a small but not negligible proportion of patients with atrial fibrillation have a concomitant supraventricular tachycardia (SVT) that acts as a trigger factor for atrial fibrillation, and these patients may benefit from a simpler ablative approach targeted only at the underlying arrhythmia elimination, without any other extra lesion (7). Ensure this tailored ablative approach is clearly the key role of a deep preliminary investigation of each patient to search for a trigger and easily-eliminable arrhythmia.

## Methods

### Study population

The study enrolled consecutive 249 patients [204 (81.9%) males] referred to Casilino Polyclinic from 2016 to 2018 for catheter ablation of paroxysmal AF with no previous documentation of synchronized SVT. In particular, the patients had recurrent episodes of paroxysmal atrial fibrillation despite antiarrhythmic therapy. The mean age of the patients was 54 ± 14 years old. Risk conditions for heart disease [hypertension, coronary artery disease (CAD), hypercholesterolemia, diabetes] were investigated for all patients by taking a medical history, cardiologic examination, and non-invasive instrumental tests (ECG and echocardiography). Specifically, were found 33 patients to be suffering from CAD (13%), 127 hypertensive (51%), 52 had hypercholesterolemia (21%), and 32 diabetics (13%). 57 patients (23%) had no heart disease (defined by the absence of CAD, valvular diseases and cardiomyopathies). ECG documented 27 (11%) atrioventricular conduction disorders, 22 (9%) left bundle branch blocks, and 52 (21%) right bundle branch blocks. Finally, echocardiography documented a mean ejection fraction (EF) value of 54.7%, a mean septal thickness of 10.8 mm, and a mean left atrium volume of 24.3 ml/m^2^. Ongoing antiarrhythmic therapy in the study population included drugs such as amiodarone, flecainide, propafenone, sotalol, beta-blockers, verapamil, and digoxin. The most widely used were beta-blockers (137, 55%), amiodarone (92, 37%), and flecainide (77, 31%). Patients were examined with electrocardiographic recordings, and if the presence of a synchronized trigger was suspected by the clinic, 24-hour ECG Holter recordings or implantable loop recorder were performed. Then, if triggers were detected, trigger-directed procedures were scheduled and they were not included in the study. While not the primary focus of the study, a comparison with a control group of paroxysmal AF patients treated in other centers (2016–2018) with a PVI-only approach was made and was resumed in Table 1.

**Table 1:** Clinical differences between study patients and control group.

	Study population (249)	Control group (52)	*P* Value
Age (years)	54 ± 14	57 ± 12	ns
Male gender (*n* %)	204 (81.9%)	42 (78%)	ns
Risk factors and heart disease aetiology			
CAD	33 (13%)	8 (15%)	ns
Hypertension	127 (51%)	32 (62%)	ns
Hypercolesterolemia	52 (21%)	15 (29%)	ns
Diabetes	32 (13%)	8 (15%)	ns
No heart disease	57 (23%)	8 (15%)	ns
Baseline ECG			
Atrioventricular conduction disorder	27 (11%)	5 (10%)	ns
LBBB	22 (9%)	4 (8%)	ns
*RBBB*	52 (21%)	11 (21%)	ns
Echocardiography			
EF (%)	54.7 ± 6.2	55.7 ± 4.8	ns
Septal thickness (mm)	10.8 ± 2.2	11.1 ± 2.1	ns
Left atrium volume (ml/m^2^)	24.3 ± 2.6	25.5 ± 3.2	ns

CAD, coronary artery disease; LBBB, left bundle branch block; RBBB, right bundle branch block; EF, mean ejection fraction.

### Electrophysiological study and ablation procedure

Any antiarrhythmic drugs were stopped for at least 5 half-lives before the procedure (for amiodarone at least 1 month before). Patients signed informed consent before undergoing the EPS and subsequent procedures; the procedure was conducted under local anesthesia and also mild sedation when required. If AF was present at the time of the EPS, electrical cardioversion was operated. If there was a recurrence of AF after 3 shocks, the study was stopped. Fortunately, this eventuality never occurred. To conduct the EPS, 3 femoral venous accesses, generally, 2 right and 1 left, were taken in the patient prepared however for AF ablation. A diagnostic decapolar catheter and a mapping/ablator catheter were deployed. The decapolar catheter was placed in the coronary sinus and the mapping catheter in the region of the para-hissian region in order to versatility manage atrial and ventricular pacing maneuvers, alternatively. Basal AH and HV intervals were recorded. Ventriculo-atrial retro-conduction was assessed with ventricular programmed stimulation in order to search for concealed accessory pathways. Atrial incremental pacing and programmed stimulation (up to three extra stimuli) were carried out in order to assess nodal-hissian antegrade conduction properties, and SVT inducibility, also by adrenergic activation with isoproterenol (with 0.5–4 μg/min dose to increase the rate of sinus rhythm by 25% compared to pre-administration). The mean procedural time for EPS was 5 min. If the trigger was identified, ablation of the trigger alone was performed; if the trigger remained undetermined, transcatheter ablation for pulmonary vein isolation (PVI) was performed. All PVIs (even in the control group with PVI-only standard approach) were performed using CARTO® V3 software with a PentaRay™ mapping catheter, a SmartTouch™ ablation catheter in a power-controlled mode (30–35 Watts) and Ablation Index module (450 in left atrium anterior wall and 330 in left atrium posterior wall, interlinear distance <6 mm) as described in other papers (8–10). Atrioventricular nodal re-entrant tachycardias (AVRNT) were treated with the slow-pathway ablation using a 4-mm, large, curved, nonirrigated ablation catheter (Blazer; Boston Scientific, Natick, MA, USA) set in the temperature-controlled mode (50 W–60°C, at least 60 s in junctional rhythm point without VA block). Atrioventricular re-entrant tachycardias (AVRT) were treated with accessory pathway ablation using non irrigated ablation catheter (Blazer) or irrigated ablation catheter (SmartTouch™) according to the judgment of the operator and based on the location (at least 60 s after accessory pathway elimination). Focal ectopic atrial tachycardias (FAT) were located initially using Kistler's electrocardiographic criteria (11) and subsequently confirmed by electroanatomical mapping during tachycardia and subsequently treated using irrigated ablation catheter (SmartTouch™) and ablation index settings as previously reported for PVI (8–10). In this group only sustained FAT were considered clinically relevant and subsequently mapped and treated.

### Follow-up

For all patients, anti-arrhythmic therapy was discontinued after the index ablation. Telemedicine was used for remote follow-up: in particular, by trans-telephonic ECG monitoring for 1 month after the procedure, recordings were taken twice daily and in any case of symptoms. Clinical evaluation, associated each time with a previous 12-lead ECG and 24-h ECG Holter, was performed at 3–6–12–24–48 months. Patients were educated to contact the Clinic if any symptoms appeared in order to add an extra visit.

### Statistical analysis

Statistical analysis was performed using SPSS statistical software (version 15.00, Chicago, IL, USA). Continuous variables were presented as means ± SD and categorical values as frequencies (%). The association between categorical variables was evaluated using Fisher's exact test. Differences in continuous variables were determined for statistical significance using independent samples of *t*-test or Mann–Whitney test. Statistical significance was defined as a two-sided probability value <0.01.

## Results

### Findings about clinical differences between patients with and without documented trigger

The EPS demonstrated the presence of a documentable trigger factor in 47 patients (18.8%) and therefore they were treated with an ablation procedure only directed to eliminate that form of SVT. The trigger was not documented in 202 patients (81.2%), who were treated with transcatheter PVI.

As shown in Table 2, patients with documented triggers were significantly younger, while the prevalence of males (however, more represented in our study) was more marked in the group without triggers. Regarding cardiovascular risk factors and the presence of heart disease, we observed that in the group of patients with documented triggers, the prevalence of CAD, hypertension, and diabetes was significantly lower, compared with the group without a documented trigger. Electrocardiographic and echocardiographic observations were also consistent with this picture. Indeed, in the group with the documented trigger we recorded on 12-lead ECG only one patient with atrioventricular conduction disturbance, no patients with left bundle branch block, and 4 patients with right bundle branch block, while in the group without documented trigger there were 26 patients with atrioventricular conduction disturbances, 22 with left bundle branch block and 48 with right bundle branch block. About echocardiography, we observed a slightly lower ejection fraction (EF) in those patients without a documented trigger (53.7% vs. 58.9%) but the difference had no statistical significance. On the other hand, there were statistically significant differences in interventricular septal thickness, which was thicker in the group without a documented trigger (11.3 ± 2.1 mm vs. 8.9 ± 2.3 mm) and there were statistically significant differences in left atrium volume, which was more dilated in the group with documented trigger than in the group without a documented trigger (25.2 ± 3.4 ml/m^2^ vs. 20.3 ± 2.2 ml/m^2^). Finally, about drug therapy, a statistically significant difference in amiodarone and digoxin use was found; amiodarone was widely used in patients without a documented trigger (44%) and scarcely in patients with documented trigger (4%), the same situation occurred for digoxin (19% vs. 4%).

**Table 2:** Findings about clinical differences between patients with and without documented triggers.

	AF with documented trigger (47)	AF without documented trigger (202)	*P* Value
Age (years)	42 ± 11	58 ± 14	<0.01
Male gender (*n* %)	32 (68%)	172 (86%)	<0.01
Risk factors and heart disease aetiology			
CAD	1 (2%)	32 (16%)	<0.01
Hypertension	5 (10%)	122 (60%)	<0.01
Hypercolesterolemia	3 (6%)	49 (24%)	<0.01
Diabetes	0	32 (16%)	<0.01
No heart disease	43 (91%)	14 (7%)	<0.01
Baseline ECG			
Atrioventricular conduction disorder	1 (2%)	26 (13%)	<0.01
LBBB	0	22 (11%)	<0.01
*RBBB*	4 (9%)	48 (24%)	<0.01
Echocardiography			
EF (%)	58.9 ± 7.2	53.7 ± 4.5	ns
Septal thickness (mm)	8.9 ± 2.3	11.3 ± 2.1	<0.01
Left atrium volume (ml/m^2^)	20.3 ± 2.2	25.2 ± 3.4	<0.01
Therapy			
Amiodarone	2 (4%)	90 (44%)	<0.01
Flecainide	12 (26%)	65 (35%)	ns
Propafenone	6 (13%)	60 (30%)	ns
Sotalol	4 (9%)	20 (10%)	ns
Beta-blockers	20 (42%)	117 (58%)	ns
Verapamil	10 (21%)	38 (19%)	ns
Digoxin	2 (4%)	38 (19%)	<0.01

CAD, coronary artery disease; LBBB, left bundle branch block; RBBB, right bundle branch block; EF, mean ejection fraction.

### Electrophysiological study and ablation results

**Figure 1:**
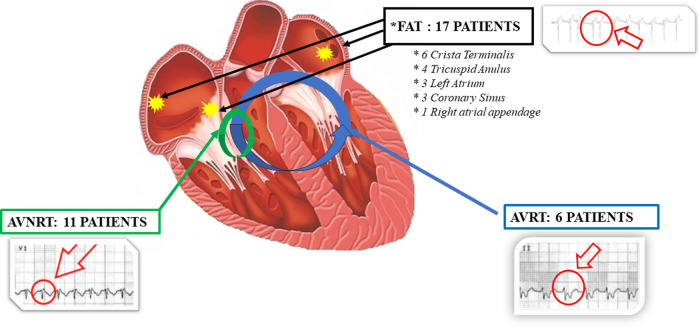
Graphic representation of the results obtained from the electrophysiological study in AF patients with documented trigger. In AVRNT group short RP interval during tachycardia in V1 lead as shown in ECG trace. In AVRT group (due to concealed Kent's bundle) long RP interval during tachycardia in II lead as shown in ECG trace. In FAT group “P on T” phenomena as shown in ECG trace. AVRNT, Atrioventricular nodal re-entrant tachycardia; AVRT, Atrioventricular re-entrant tachycardias; FAT, focal atrial tachycardia.

The electrophysiological study results were (Figure 1): 11 patients had AVNRT, 6 patients had AVRT due to concealed Kent's bundle, 17 patients had FAT that, more precisely speaking, in 3 patients originated in the left atrium outside the pulmonary veins, in 6 patients from the crista terminalis, in 3 patients from the coronary sinus, in 4 others from the tricuspid annulus and in one patient from the right appendage. 13 patients had the trigger in a single arrhythmogenic pulmonary vein. However in two patients, there were two arrhythmogenic pulmonary veins, and in these cases, complete PVI was performed).

Subsequently, the ablation procedure was performed (classical PVI or trigger-targeted ablation according to whether the patient belonged to one of the two groups) and it was successful in all patients. The ablation procedure was guided by the ablation index (AI) in PVI patients and in a patient with focal ectopic atrial tachycardia from a single pulmonary vein. There were no serious complications in either group, only 5 groin hematomas needing no treatment were detected in the group undergoing PVI. Mean ablation time in PVI group was 30 ± 11 min, 4 ± 2 in triggered ablation group, 31 ± 9 in control group. Fluoroscopy times observed were: 4 ± 1.6 min in the 47 SVT patients, 7 ± 2 min in PVI patients and 6 ± 2 in control group.

### Follow-up

In the PVI-treated group, 121 (60%) patients remained free from atrial fibrillation recurrences at 32 ± 18 months follow-up, 96 (47%) patients needed to resume antiarrhythmic therapy, and 46 (23%) patients underwent a second PVI procedure. In the group with identified triggers, in 32 ± 18 months mean follow-up there were 4 patients (8.5%) who had atrial fibrillation recurrences (one patient treated for AVNRT, 2 for FAT, and another one had a single arrhythmogenic pulmonary vein at the previous EPS); excluding these 4 patients, no other patients needed to take any antiarrhythmic therapy.

### AF-free survival

**Figure 2:**
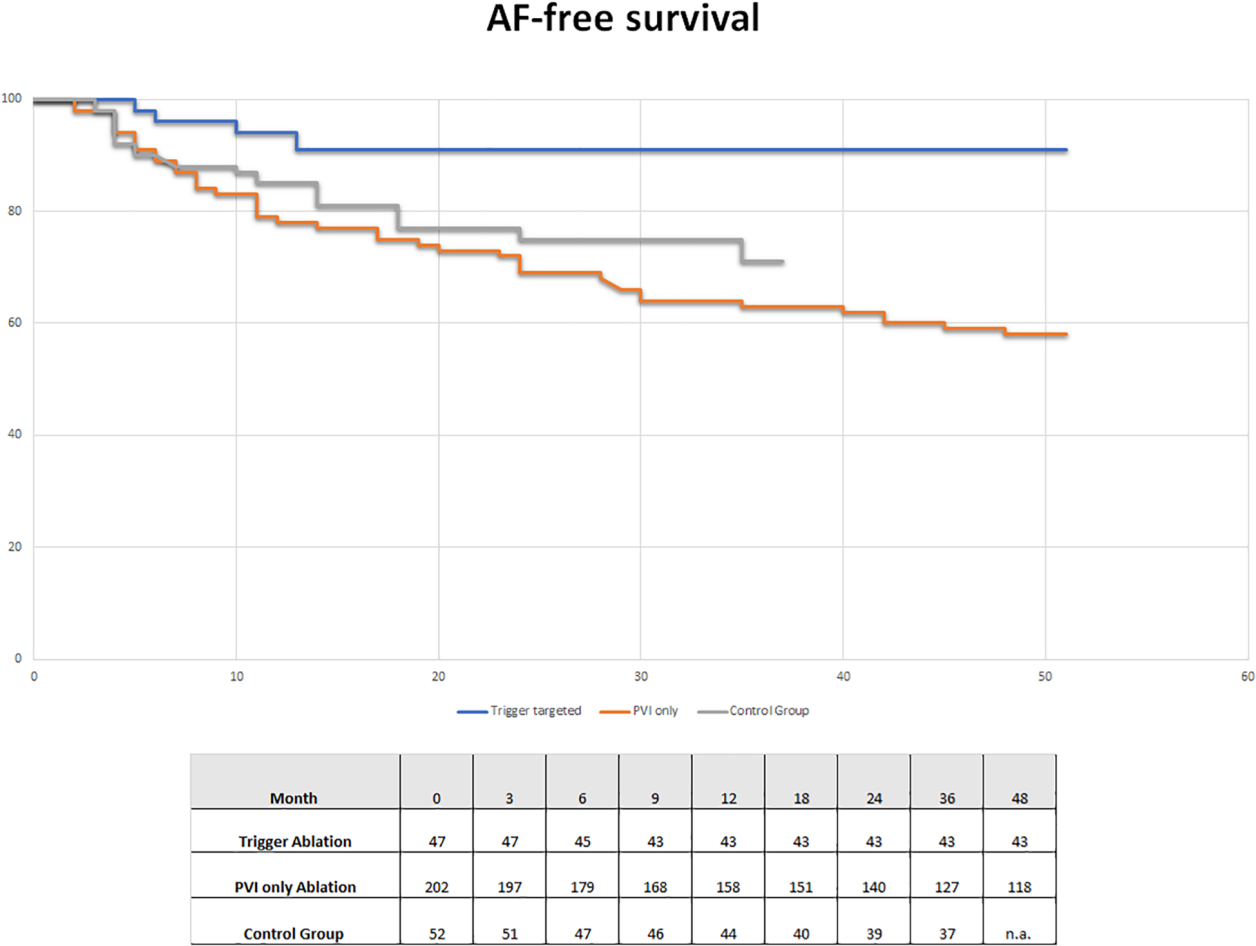
AF-free survival group in trigger ablation (blue line) and PVI-only (red line) patients of our study and in the control group (grey line) treated with a PVI-only approach without trigger research.

Figure 2 shows the AF-free survival curves of the two groups (PVI-only group and trigger-targeted ablation) also compared with the control group of patients treated using a PVI-only approach. At 2 years follow-up non-significant differences were observed between our PVI-only group and the control group, but a favourable trend is evident in the control group. On the other hand, the advantage of trigger-targeted ablation is substantial, which is confirmed in the long-term follow-up.

## Discussion

### Main findings

In our study, we observed that in an unselected population of patients who were candidates for PVI to treat paroxysmal atrial fibrillation, as many as 18.8% had a definite trigger mechanism that could be documented during an electrophysiological study, which could be represented by the presence of atrioventricular nodal re-entrant tachycardia (AVNRT), atrioventricular re-entrant tachycardia (AVRT), focal ectopic atrial tachycardia (FAT), including some from a single arrhythmogenic pulmonary vein originating tachycardia. Patients belonging to this sub-population were more likely to be younger and with a lower incidence of structural heart disease, cardiovascular risk factors, and AV and IV conduction disturbances. These patients were treated with a targeted ablation procedure exclusively at the trigger mechanism identified in the EPS; the safety and long-term efficacy (91.5%) of such ablation procedures were shown to be excellent, confirmed by an average follow-up time of 32 ± 18 months in which the recurrence rates of atrial fibrillation were very low and almost no patients (only 4 out of 47) had to resume rhythm control therapy or repeat the ablation procedure.

### Tailoring the ablation

PVI is the cornerstone of the transcatheter ablation procedure for atrial fibrillation. The reason is that the pulmonary veins represent the most common “trigger site” for arrhythmogenic activity, as first demonstrated by Haissaguerre (2); in addition, the lesions produced during PVI also affect other less common trigger sites (Marshall's vein and ligament, posterior atrial wall of the left atrium) and according to some authors could also act on the substrate, due to the elimination of tissue that can host reentry circuits, as well as on the disruption of sympathetic and parasympathetic innervation (3). However, the efficacy outcomes in both the short and long term and the safety profile of PVI, which are not yet satisfactory as demonstrated by the failure to decrease complication rates over the years (12, 13), must force the scientific community to seek in every way to increase the performance of the transcatheter ablation procedure.

From all these considerations it seems clear to us that we can improve the outcomes of ablations for atrial fibrillation only by working from all points of view (organizational, technological, and pathophysiological-procedural) on the daily clinical practice of atrial fibrillation treatment. Our study focused precisely on the pathophysiological aspect underlying the ablation procedure, starting from the consideration that it is true that the pulmonary veins represent the main site stimulating arrhythmogenic activity underlying atrial fibrillation, but it is equally true that they are not the exclusive site and that other supraventricular tachycardia may trigger atrial fibrillation. In 2010 it was shown that in clinical practice it also happens that patients in whom only atrial fibrillation is known to be present (*either paroxysmal or persistent*) and who are candidates for classical PVI, when subjected to electrophysiological study as many as 10.1% (26 out of 257 total patients) had undiagnosed atrial focal tachycardia, AVNRT or AVRT; treating these patients with ablation targeted only at the elimination of supraventricular tachycardia showed excellent efficacy results, with only two AF relapses in the follow-up time of 21 ± 11 months (7). Previously other studies had already evaluated the efficacy of catheter ablation for AVRT and AVRNT and subsequent AF recurrences, in patients suffering from the latter. In 2005, Wang and colleagues demonstrated that out of 33 patients with AVRT and atrial fibrillation in whom an ablation procedure had been performed on the atrioventricular accessory pathway, AF recurrence was observed in 4 patients. These 4 patients had a much higher mean age (64 ± 5 vs. 40 ± 11) than the group without AF recurrence (14). It is therefore evident that it is necessary to better understand, in presence of patients with supraventricular tachycardia, those who can be healed by eliminating the tachycardia, identifying the main risk factors for atrial fibrillation relapse. The importance of age as a risk factor for atrial fibrillation relapse after ablation of supraventricular tachycardia was confirmed by a meta-analysis published in 2020 (15).

Although age is considered the only independent risk factor for AF relapse in this meta-analysis, it is reasonable to think that younger and also healthier hearts are more likely to be free of mechanisms (i.e., structural remodeling) that, depending or not depending of the presence of SVT, may promote the relapse of AF. In our study, of the 47 patients with documented triggers, the mean age was 42 years, there were no diabetics, and as many as 43 out of 47 had no structural heart disease; the percentage of hypertensives was also low (10% of total).

One of the new findings of our study is the treatment of atrial fibrillation triggered by a single arrhythmogenic pulmonary vein, which consisted of isolation of only the affected vein. This case involved 13 patients out of 249 total (5.2%), which is certainly not an insignificant number. What is most interesting is the long-term efficacy of the procedure: of these 13 patients only one needed to take antiarrhythmic drugs at the 32-month follow-up. Thus, the treatment had a very high (92.3%) long-term efficacy. Few reports are available regarding the ideal treatment for patients with a single arrhythmogenic vein. In one case study, published in 2004, a single arrhythmogenic vein inducing high ventricular rate AF was treated with circumferential ablation at the responsible pulmonary vein ostium thus favorably resolving the condition (16). Other studies have demonstrated efficacy in randomized trials of such treatment around 75% (17) with a well-known consistent role of triggering vein for atrial fibrillation recurrence due to its long-term reconnection (18). However, our study was carried out in an era in which the use of lesion parameters such as Ablation index demonstrated high efficacy pulmonary veins isolation, even in the long term (9), thus justifying the high efficacy of treatment in our series. The characterization of the vein responsible for the arrhythmia in these patients allows it to be treated exclusively, in order to avoid additional lesions in the left atrium, with a reduction in possible complications, procedural times and iatrogenic arrhythmias induced by PVI (i.e., atypical flutter) (19, 20).

However, a challenge is to define those patients who can really be healed by a target ablation. Greater attention to the clinic is necessary: in our series of 47 patients who had never been diagnosed with paroxysmal supraventricular tachycardia, 12 reported episodes of palpitations with a rhythmic pulse and a “change in the symptom palpitations which then became irregular”. Thus, in this case, medical history was a valuable means of understanding the diagnosis.

### It's a matter of time

Recently EAST-AFNET 4 trial (21) demonstrated the superiority of early rhythm control strategy vs. usual care (rate control) in an elderly population with heart disease. The reasons for the difference in outcome between the old trials [including the famous AFFIRM (22)] and the new ones are various, including certainly the use in the more recent studies of PVI as a method for rhythm control, but great importance is also attributable to the “time factor”: in EAST-AFNET 4 the rhythm control intervention was always instated early, within 12 months of the diagnosis of atrial fibrillation. From these considerations may follow the importance of rhythm control strategy once the decision has been made to pursue this therapeutic approach. The question then arises spontaneously: is our “tailored approach” time-consuming? No, it's not. The patients involved in our study were already candidates for pulmonary vein ablation and simply underwent a pre-ablation electrophysiological study, thus not delaying the ablation procedure in any way.

### Comment on our results

In our study of 249 PVI candidate patients for paroxysmal atrial fibrillation, we conducted an electrophysiological study looking for a subpopulation in which supraventricular tachycardia was the trigger factor of atrial fibrillation. The young age and the good health conditions of many patients, the clinical condition of paroxysmal [and non-persistent, different from the 2010 previous paper (7)] atrial fibrillation as well as a suggestive clinic in 12 patients (who reported episodes of palpitations with a rhythmic pulse that then became irregular) suggested that it was probable to find a good percentage of patients with supraventricular tachycardia. On these patients (in which we also included 13 patients with tachycardia originating from a single pulmonary vein, PVT) we conducted an ablation aimed exclusively at the elimination of the trigger. Even if the transition from supraventricular tachycardia to atrial fibrillation was observed in the electrophysiological study in only a part of the patients (44% of AVNRT, 50% of AVRT, 46% of FAT, and 54% of PVT), nevertheless the good efficacy final tells us that patients with a high *a priori* possibility of having a form of atrial fibrillation secondary to supraventricular tachycardia have been well identified. A loop recorder was not implanted in the follow-up and this could be one of the limitations of the study considering that this instrument has greater sensitivity in identifying recurrences of atrial fibrillation (23), however, we believe that in our specific study population this is not a great limitation as our patients with supraventricular tachycardia were strongly symptomatic and would have reported the return of symptoms during the trans-telephonic monitoring or the periodic ambulatory visit. In terms of comparison between the standard PVI strategy and our approach, a limitation of the study was the comparison with a control group of paroxysmal AF patients not treated in the same center with different operators, a choice made in order to have a group of patients who were homogenous in terms of clinical characteristics and procedural aspects [PVI-only approach with Ablation Index module (8–10)]. The favorable trend of the control group in terms of survival (albeit with a non-equal length of follow-up) can be justified by the fact that a PVI-only approach also includes the ablation of patients with FAT originating from a single pulmonary vein, which in our study belong to the triggered ablation group.

Another limit of our study is the sample size, which, however, if adequately increased in a polycentric study could lead to a poly parametric score (clinical-anamnestic-electrocardiographic and echocardiographic) capable of predicting subjects with a high probability of SVT in which a pre-PVI EPS study could be mandatory. In fact, based on our study, we can indicate younger subjects, without ECG and Echocardiographic alterations, as at risk for SVT-triggered AF without however being able to indicate precise cutoffs.

## Conclusions

A subpopulation can be selected from the candidates for PVI that can be targeted for highly effective and low-risk ablations, characterized by short procedural times and minimal lesion load required. The efficacy and safety of the procedures are supported by long-term follow-up. We need further studies to know how better and earlier identify patients who can be cured by tailored ablations and at the same time to understand in which patients there are major risk factors for atrial fibrillation recurrence after a tailored approach or, conversely, if simply the age can be considered the only marker of the risk of AF recurrence.

## Data Availability

The raw data supporting the conclusions of this article will be made available by the authors, without undue reservation.
